# Supplementary material for: Rapid HPLC-ESI-MS/MS Analysis of Neurotransmitters in the Brain Tissue of Alzheimer’s Disease Rats before and after Oral Administration of Xanthoceras sorbifolia Bunge
Source: Molecules. 2018 Nov 28;23(12):3111. doi: 10.3390/molecules23123111 (PMC6321314; doi:10.3390/molecules23123111)
Supplement: Supplementary file 1 [file molecules-23-03111-s001.pdf]

Supplementary Material

Rapid analysis of neurotransmitters in the brain tissue of Alzheimer's disease rats before and after oral administration of Xanthoceras sorbifolia Bunge by HPLC-ESI-MS/MS

Zheng Sun[1], Qing Li[1] and Kaishun Bi[1,*]

[1] National and Local Joint Engineering Laboratory for Key Technology of Chinese Material Medica Quality Control, School of Pharmacy, Shenyang Pharmaceutical University, 103 Wenhua Rd.,
Shenyang 110016, China; sunzheng024@163.com (Z.S.); lqyxm@hotmail.com (Q.L.)

* Correspondence: kaishunbi.syphu@gmail.com; Tel.: +86-24-4352-0589

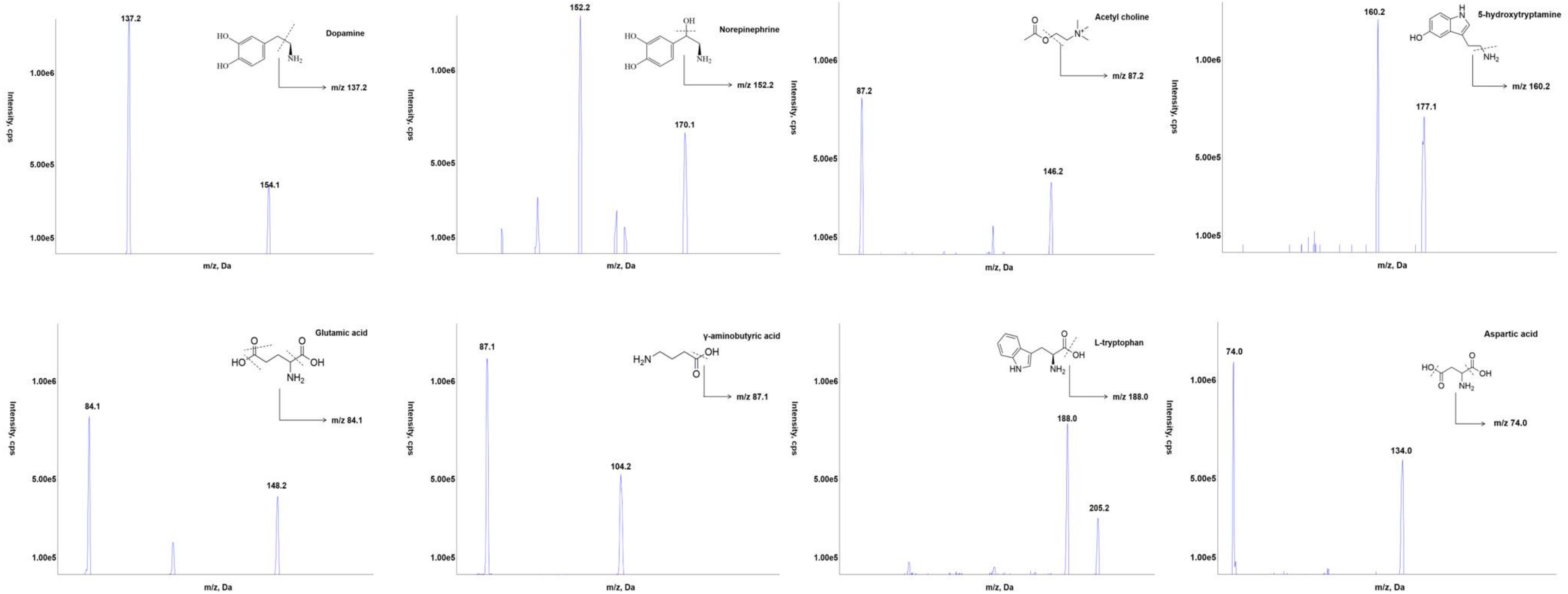

Supplementary Figure 1 Chemical structures and full scan of product ion spectrums from Dopamine, Norepinephrine, Acetyl choline, 5-hydroxytryptamine, Glutamic acid, γ-aminobutyric acid, L-tryptophan and Aspartic acid.

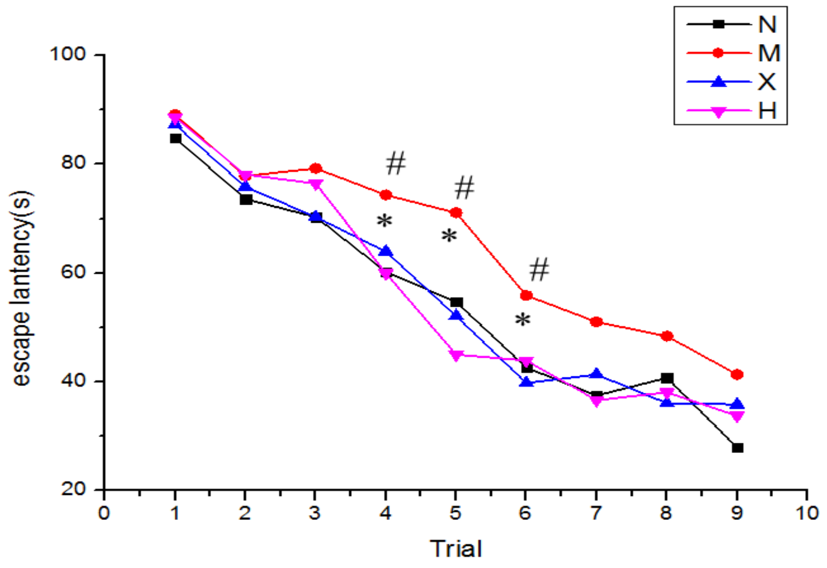

Supplementary Figure 2 Effect of Xanthoceras sorbifolia Bunge on escape latency in rats with AD in the directional swimming test of 5 days.

N: control group; M: model group; X: Xanthoceras sorbifolia Bunge group; H: huperzine group.

#p < 0.05, compared with N group; *p < 0.05, compared with X group.

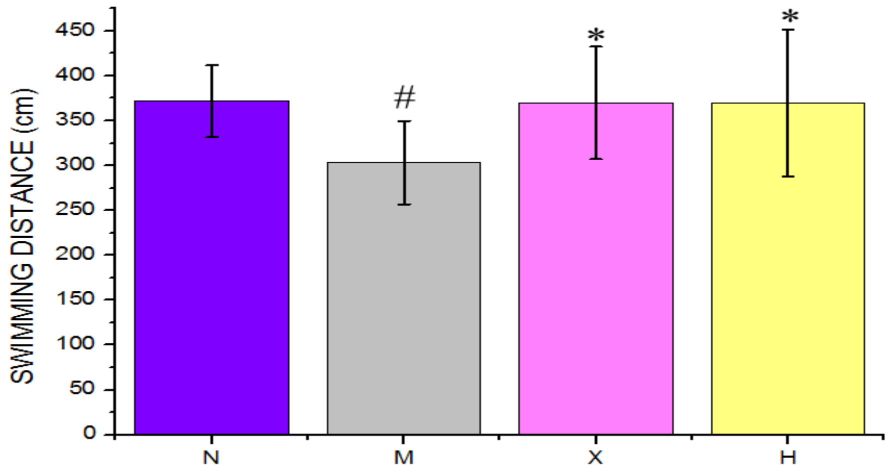

Supplementary Figure 3 The effect of Xanthoceras sorbifolia Bunge on the swimming distance in the target quadrant in model rats in probe test.

N: control group; M: model group; X: Xanthoceras sorbifolia Bunge group; H: huperzine group.

#p < 0.05 compared with control group; *p < 0.05, compared with model group.

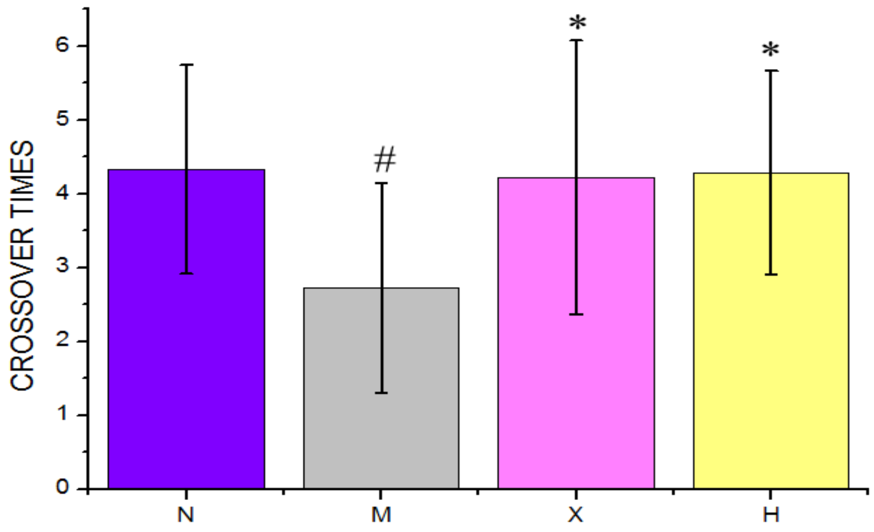

Supplementary Figure 4 The effect of Xanthoceras sorbifolia Bunge on the number of across the platform in model rats in probe test.

N: control group; M: model group; X: Xanthoceras sorbifolia Bunge group; H: huperzine group.

#$p < 0.05$ compared with control group; *$p < 0.05$, compared with model group.
